# Long-term prescribed drug use in stage I–III rectal cancer patients in Sweden, with a focus on bowel-regulating drugs after surgical and oncological treatment

**DOI:** 10.1007/s11764-024-01548-9

**Published:** 2024-02-06

**Authors:** Sol Erika Boman, Stina Fuentes, Caroline Nordenvall, Anna Martling, Lingjing Chen, Ingrid Glimelius, Martin Neovius, Karin E. Smedby, Sandra Eloranta

**Affiliations:** 1 Division of Clinical Epidemiology, Department of Medicine Solna, Karolinska Institutet, Karolinska University Hospital, 17176 Stockholm, Sweden; 2 Department of Molecular Medicine and Surgery, Karolinska Institutet, Karolinska University Hospital, 17176 Stockholm, Sweden; 3 Division of Gastroenterology, Medical Unit Gastroenterology, Dermatovenereology and Rheumatology, Karolinska University Hospital, Stockholm, Sweden; 4 Department of Immunology, Genetics and Pathology, Cancer Precision Medicine, Uppsala University, 75185 Uppsala, Sweden

**Keywords:** Rectal cancer, Drug use, Survivorship, Epidemiology, Population-based

## Abstract

**Purpose:**

To describe long-term prescribed drug use after rectal cancer treatment.

**Methods:**

We identified 12,871 rectal cancer patients without distant metastasis between 2005 and 2016 and 64,341 matched population comparators using CRCBaSe (a Swedish nationwide register linkage of colorectal cancer patients). Mean defined daily doses (DDDs) of drug dispensing during relapse-free follow-up were calculated by Anatomical Therapeutic Chemical drug categories. Incidence rate ratios (IRRs) and 95% confidence intervals (CIs) from negative binomial regression were used to compare drug dispensing between patients and comparators.

**Results:**

The overall pattern of drug dispensing was similar among cancer survivors and comparators, although patients had higher mean DDDs of drugs regulating the digestive system. Excess dispensing of drugs for constipation (IRR, 3.35; 95% CI, 3.12–3.61), diarrhea (IRR, 6.43; 95% CI, 5.72–7.22), functional gastrointestinal disorders (IRR, 3.78; 95% CI, 3.15–4.54), and vitamin and mineral supplements (IRR, 1.37; 95% CI, 1.24–1.50) was observed up to 10 years after surgery. Treatment with Hartmann’s procedure was associated with higher dispensing rates of digestive drugs compared to surgery with anterior resection and abdominoperineal resection but the association was attributed to higher use of diabetic drugs. Additionally, excess digestive drug dispensing was associated with more advanced cancer stage but not with (chemo)radiotherapy treatment.

**Conclusions:**

Excess drug use after rectal cancer is primarily driven by bowel-regulating drugs and is not modified by surgical or oncological treatment.

**Implications for Cancer Survivors:**

The excess use of bowel-regulating drugs after rectal cancer indicated long-standing postsurgical gastrointestinal morbidity and need of prophylaxis. Reassuringly, no excess use of other drug classes was noted long term.

**Supplementary Information:**

The online version contains supplementary material available at 10.1007/s11764-024-01548-9.

## Introduction

Rectal cancer survivors have reported an array of symptoms, including urogenital dysfunction, bowel dysfunction, pelvic insufficiency fractures, peripheral neuropathy, and sexual dysfunction [1]. Worsened social well-being, especially among young (aged < 75 years) and female patients, has also been reported among long-term (> 10 years) rectal cancer survivors with an ostomy from surgery [2, 3]. Moreover, impaired quality of life from both physical and psychological symptoms has been reported [4], with anxiety, depressive symptoms, or cancer-related side effects, such as pain, hot flushes, and nausea being indicators for treatment with anti-depressants [5]. Rectal cancer is also associated with a long-term risk of work loss, including disability pension and sick leave, following abdominal surgery among working-age rectal cancer survivors compared to the general population, with underlying reasons likely to be multifactorial [6–10].

Postsurgical drug use has not previously been investigated systematically in long-term rectal cancer survivors. Using drug dispensing as a proxy for drug use, this study aims to characterize the panorama of health problems that may be associated with long-term side effects, reduced work ability, and decreased quality of life among rectal cancer patients. In addition to overall drug use up to 10 years after curative surgical treatment, we also studied the dispensing of drugs that regulate the digestive system in relation to elapsed time since surgery and given treatment, including type of surgery and oncological treatment.

## Methods

The Colorectal Cancer Database [11] (CRCBaSe) was used to identify rectal cancer patients diagnosed in Sweden between July 1, 2005, and December 31, 2016, and to sample comparators from the general population. The matched cohort was followed up for all drugs dispensed as recorded in the Swedish Prescribed Drug Register.

The study was performed in line with the principles of the Declaration of Helsinki. The study was approved by the Regional Ethics Review Board in Stockholm (approval nos. 2014/71–31, 2018/328–32, and 2021–00342).

### Study population

#### Rectal cancer patients

Out of 21,355 patients receiving their first rectal cancer diagnoses during the study period, exclusions were made for patients with stage IV or unknown stage cancers (*n* = 7500), patients not treated with curative abdominal surgery (*n* = 872), and patients with missing or erroneous information on surgical procedure or cancer relapse dates (*n* = 110). Two patients were further excluded due to a lack of eligible comparators or follow-up time due to death at the inclusion date. The remaining patient cohort consisted of 12,871 individuals, for which clinical information on pathological TNM stage (I–III), operation type (anterior resection, abdominoperineal resection, Hartmann’s procedure), oncological treatment (combinations of neoadjuvant and adjuvant chemotherapy (CT) and radiotherapy (RT)), and cancer relapse were obtained from the register.

#### Matched comparators

CRCBaSe consists of approximately 77,000 colorectal cancer patients and 462,000 comparators (1:6) matched on (legal) sex, age, and region. For the purposes of this study, we aimed to resample five comparators for each rectal cancer patient in our cohort from within CRCBaSe while matching on sex, birth year (age), vital status, and an unweighted version of the Charlson Comorbidity Index (CCI). The unweighted CCI (categorized as 0, 1, or > 1 comorbidity) was chosen instead of the weighted version of the CCI (in which included diseases are attributed a weight based on their relative mortality) to capture confounding by drug prescription indication. The CCI was calculated using inpatient and non-primary care outpatient records up to 5 years before the rectal cancer diagnosis date from the National Patient Register, as well as records of previous cancer diagnoses from the Swedish Cancer Register (SCR) up to 10 years before the diagnosis date [12]. The final matched cohort consisted of 12,871 patients and 64,341 comparators. Using this matching criterion, there were five rectal cancer patients for which we were unable to sample a full comparator set.

Information on the highest attained education (≤ 9, 10–12, or > 12 years of education, corresponding to primary, secondary, and university studies in Sweden) was subsequently linked to our matched cohort from the Longitudinal Integrated Database for Health Insurance and Labor Market Studies (LISA), maintained by Statistics Sweden since 1990. Records of death and emigration were linked from the Cause of Death Register and the Total Population Register, respectively.

### Drug use and defined daily dose (DDD)

Drug dispensing data were obtained from the Prescribed Drug Register (PDR). The register was established in July 2005 and contains information on all dispensed prescribed drugs in the Swedish population [13, 14]. The drugs are classified according to the Anatomical Therapeutic Chemical (ATC) classification system, which consists of 14 main anatomical groups and further therapeutic/pharmacological/chemical subgroups under each main group [15]. In this study, anatomical (first), therapeutic (second), and therapeutic/pharmacological (third) level information were used to extract and classify drug use in the cohort:

- A (alimentary/digestive tract and metabolism, e.g., drugs for peptic ulcer)
- B (blood and blood-forming organs, e.g., anti-thrombotic agents and anti-hemorrhagics)
- C (cardiovascular system, e.g., lipid-modifying agents and beta-blockers)
- N (nervous system, e.g., hypnotics/sedatives, opioids, and anti-depressants)
- Others, including D (dermatologicals), G (genitourinary system and sex hormones), H (systemic hormonal preparations, excl. sex hormones and insulins), J (anti-infectives for systemic use), L (anti-neoplastic and immunomodulating agents), M (musculo-skeletal system), P (anti-parasitic products, insecticides, and repellents), R (respiratory system), and S (sensory organs)

ATC codes relating to symptoms in the digestive system (alimentary tract and metabolism), possibly associated with rectal cancer or its treatment, were also studied by subcategory. These categories included A02 (drug for acid-related disorders), A03 (drugs for functional gastrointestinal disorders), A06 (drugs for constipation), A07 (anti-diarrheal, intestinal anti-inflammatory/anti-infective agents), A10 (drugs used in diabetes), A11 (vitamins), and A12 (mineral supplements).

The defined daily dose (DDD) is a construct used to facilitate a standardized comparison of drug usage. It was defined by the World Health Organization as the assumed average maintenance dose per day for a drug used for its main indication in adults [16]. In this study, the DDDs of dispensed drugs were calculated by multiplying the number of packages by the DDD per package for every dispensed prescription recorded in the PDR. It was assumed that all prescribed doses were consumed. Among censored individuals, it was assumed that the individual consumed one daily dose per day of their last prescription of each drug until the day of censoring.

### Statistical analyses

The cohort was followed from the date of curative abdominal surgery (matching date for comparators) until the date of emigration, death, or Dec 31, 2017, whichever came first. Rectal cancer patients were also censored at the date of any cancer relapse, and comparators were censored if they were diagnosed with colorectal cancer during follow-up.

Demographic and clinical characteristics of patients and comparators were summarized and compared using *χ*^2^-tests. The mean DDD per year of follow-up (as well as for reference 2 years before the start of follow-up) was calculated for overall drug use, per ATC category, and for subcategories of drugs used to regulate the digestive system. The mean was calculated as the ratio between the total DDDs per year and the total number of person-years contributed in that time period. In addition, annual DDD levels were grouped according to 0, > 0–180 (up to 6 months), > 180–360 (6 months–1 year), and > 360 (1 year) to form categories for describing differences in the load of DDDs between the patients and the comparators.

To model the rate of dispensed digestive drugs, we estimated incidence rate ratios (IRRs) of DDDs for this drug category using a negative binomial model [17]. To relax the assumption of a constant baseline rate of DDDs during follow-up, each individual’s total follow-up was split into 1-year time bands that were included as covariates in the model together with an offset term containing the individual person-time at risk during that year. To account for the dependent data structure introduced by the time splitting (whereby an individual may contribute DDDs to multiple time bands), the models were further adjusted for intraindividual correlation using a robust estimator of the standard error [18]. Further included in the models were the case/comparator indicator, age at matching date, sex, CCI, education level, and calendar period. Temporal trends in dispensing patterns of drugs that regulate the digestive system were assessed formally via interaction analyses (between case and comparator status) and calendar period of diagnosis (2005–2008, 2009–2012, 2013–2016) where the significance of the interaction terms was tested using likelihood ratio tests. To study non-proportional hazards (i.e., if the relative rate of DDDs, comparing cases to comparators, varied across year of follow-up), interaction terms between the case and comparator indicator, and the 1-year time bands representing follow-up time were included in a second set of regression models.

The association between clinical characteristics, including treatment types (surgical and oncological treatments) and stage, and the rate of digestive drug use was studied in a third set of negative binomial regression models. These analyses were restricted to the patient population, limited to the first 5 years after surgery, and included the same adjustment factors as described above. As sensitivity analyses, these models were also refitted without including drugs used to treat diabetes when calculating the DDDs.

In all models, a complete case approach to handling missing data was used.

Data analysis was performed using SAS (version 9.4, SAS Institute Inc., Cary, NC, USA), STATA (StataCorp, release 14.1. College Station, TX, USA), and the R statistical software.

## Results

The median age at diagnosis or matching date (for comparators) was 69 years. Sixty percent were male, and the median follow-up time was 3.8 years (range, 0–10) for patients and 5.4 years (range, 0–10) for comparators (Table 1). Due to matching, the distribution of sex, age, CCI, and calendar period of surgery/matching was identical between the groups. In terms of pathological stage distribution, 32% of the patients were in stages 0–I, 31% in stage II, and 37% in stage III. The majority were operated with anterior resection (53%), followed by abdominoperineal resection (36%) and then Hartmann’s procedure (11%). Neoadjuvant RT was administered to 67% of patients, and a total of 14% of patients received RT alongside adjuvant CT, whereas 53% of patients received RT without adjuvant CT. Overall, 16.2% of the patients received neoadjuvant RT combined with CT (with or without adjuvant CT). Further, 4% of the patients were given adjuvant CT with no neoadjuvant treatment and 28% received no (neo)adjuvant treatment. Local or distant recurrence occurred in 20% of the patients, whereby they were censored from the analysis.

**Table 1:** Demographical and clinical characteristics of rectal cancer patients diagnosed in Sweden between 2005 and 2016 and their matched population comparators

	Rectal cancer patients Patients N (%)	Matched comparators N (%)	*P-*value^4^ *P*-value
*N*	12,871	64,341	
Median age at diagnosis	69	69	
Median follow-up years (range)	3.84 (0–10)	5.38 (0–10)	
Sex			1.000
Male	7718 (60.0%)	38,581 (60.0%)	
Female	5153 (40.0%)	25,760 (40.0%)	
Age at surgery^1^			1.000
< 50 years	714 (5.5%)	3556 (5.5%)	
50–59 years	1780 (13.8%)	8900 (13.8%)	
60–69 years	4020 (31.2%)	20,100 (31.2%)	
70–79 years	4291 (33.3%)	21,455 (33.3%)	
> 80 years	2066 (16.1%)	10,330 (16.1%)	
Calendar period of surgery^1^			1.000
2005–2008	3654 (28.4%)	18,268 (28.4%)	
2009–2012	4487 (34.9%)	22,428 (34.9%)	
2013–2016	4730 (36.7%)	23,645 (36.7%)	
Education level			0.008
Less than 9 years	4659 (36.2%)	22,844 (35.5%)	
9–12 years	5202 (40.4%)	25,462 (39.6%)	
> 12 years	2878 (22.4%)	15,150 (23.5%)	
Missing	132 (1.0%)	885 (1.4%)	
Charlson Comorbidity Index^2^			0.997
0	9273 (72.0%)	46,365 (72.1%)	
1	2719 (21.1%)	13,595 (21.1%)	
> 1	879 (6.8%)	4381 (6.8%)	
Surgery types			
Anterior resection	6804 (52.9%)	NA	-
Abdominoperineal resection	4607 (35.8%)	NA	-
Hartmann’s procedure	1460 (11.3%)	NA	-
Oncological treatment			
Neoadjuvant RT, no adjuvant CT	6817 (53.0%)	NA	-
Neoadjuvant RT, adjuvant CT	1813 (14.1%)	NA	-
No neoadjuvant treatment, adjuvant CT	560 (4.4%)	NA	-
No treatment	3607 (28.0%)	NA	-
Neoadjuvant CT only	37 (0.3%)	NA	-
Neoadjuvant CT, no neoadjuvant RT, adjuvant CT	20 (0.2%)	NA	-
Neoadjuvant info missing (CT/RT)	17 (0.1%)	NA	-
Pathologic stage			
Stages 0–I^3^	4111 (31.9%)	NA	-
Stage II	3980 (30.9%)	NA	-
Stage III	4780 (37.1%)	NA	-
Relapse within 10 years			
Yes	2627 (20.4%)	NA	-

*RT* radiotherapy, *CT* chemotherapy

^1^Matching date for comparators

^2^Unweighted

^3^Of which 354 had stage classified as 0

^4^Chi-square test of frequencies between patient and comparator groups

### Drug use patterns among cases and comparators

**Fig. 1:**
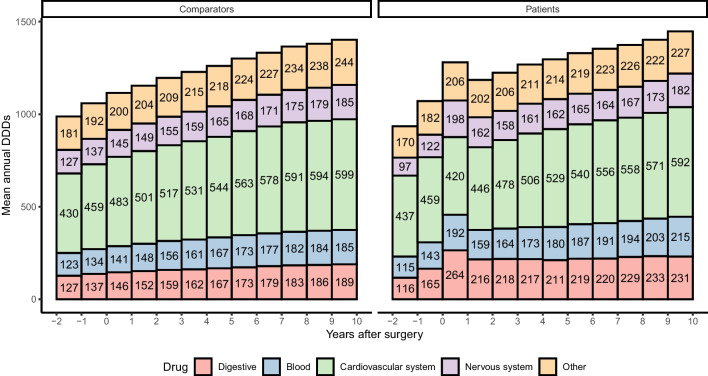
The mean defined daily dose (DDD) prescribed annually to rectal cancer patients diagnosed between 2005 and 2016 and their matched comparators. Results are presented by main Anatomical Therapeutic Chemical (ATC) classification by year of follow-up in relation to the date of curative abdominal surgery, as well as for the 2 years preceding the surgery (for reference)

The total DDDs of dispensed drugs are presented descriptively by main drug category in Fig. 1. Two years prior to surgery (index date), there was no notable difference between patients and comparators, with the exception of nervous system drugs (i.e., hypnotics/sedatives and anti-depressants), for which comparators had slightly elevated use compared to patients.

In the diagnostic year, patients were generally prescribed more drugs than comparators. After this initial peak, the overall mean annual DDDs also remained higher than for comparators throughout the 10-year follow-up. The excess drug use was mainly represented by the digestive drug category, in which the patients’ mean annual DDDs during follow-up ranged from 211 to 264 (min, max), whereas levels among comparators ranged from 146 to 189. For nervous system drugs and blood and blood-forming organ drugs (e.g., anti-thrombotic agents, anti-hemorrhagic, anti-anemic preparations, blood substitutes, and perfusion solutions), patients had higher mean annual DDDs than comparators during the diagnostic year after which the difference attenuated, particularly for the former drug category. With respect to circulation system drugs, a lower mean DDD was observed among patients than in comparators during the entire follow-up.

### Drug utilization patterns with a focus on bowel movement–regulating drugs

**Fig. 2:**
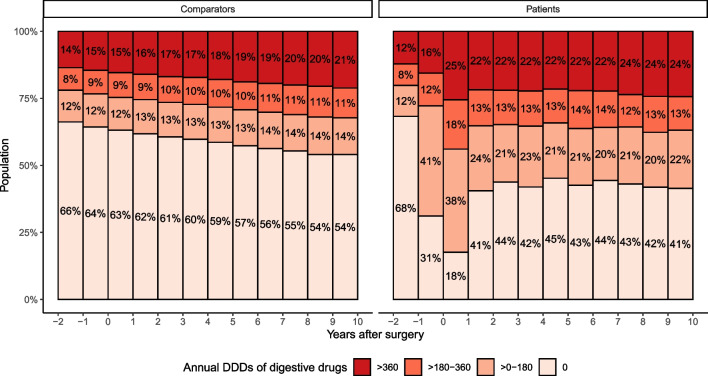
The proportion of rectal cancer patients and matched comparators that were prescribed 0, 0–180, 180–360, or > 360 defined daily doses (DDDs) annually of drugs relating to the digestive system (ATC main classification “A”). Results are presented by year of follow-up in relation to the date of curative abdominal surgery, as well as for the 2 years preceding the surgery (for reference)

The dispensing of digestive drugs was close to identical for patients and comparators 2 years before the cancer diagnosis date (Fig. 2).

**Fig. 3:**
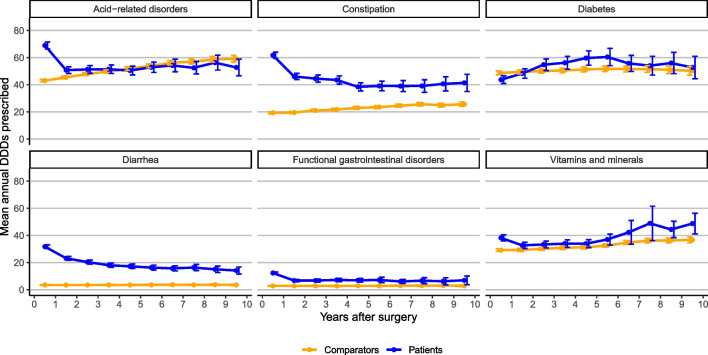
The mean annual defined daily doses (DDDs), including 95% confidence intervals, of drugs relating to the digestive system (ATC main classification “A”) prescribed to rectal cancer patients diagnosed between 2005 and 2016 and their matched comparators. Results are presented by therapeutic subgroup of digestive drugs and by year of follow-up in relation to the date of curative abdominal surgery

During the year that preceded the diagnosis, 69% of the patients were prescribed at least one digestive drug (36% among comparators), and during the diagnostic year, the corresponding proportion was 82% (37% among comparators). This proportion gradually shifted toward approximately 60% among the patients for the remainder of the follow-up up to 10 years. Moreover, approximately 20–25% of the patients used digestive drugs on a daily basis (annual DDDs > 360) throughout follow-up. For comparators, the proportion of daily users was approximately 15–20%. When further investigating digestive drug subcategories (Table 2, Fig. 3), adjusted models showed an overall higher dispensing rate among the rectal cancer survivors than in comparators for all subcategories except diabetes drugs.

**Table 2:** Incidence rate ratio (IRR) of defined daily doses, comparing rectal cancer patients diagnosed in Sweden between 2005 and 2016 and their matched comparators. A negative binomial regression model was estimated stratified by the therapeutic subgroup of digestive drugs and performed on a time-splitted dataset to allow a piecewise linear (annual follow-up bands) baseline prescription rate

	Therapeutic subgroup of drugs used for the digestive system					
	Acid-related disorders IRR (95% CI)	Constipation IRR (95% CI)	Diabetes IRR (95% CI)	Diarrhea IRR (95% CI)	Functional gastrointestinal disorders IRR (95% CI)	Vitamins and minerals IRR (95% CI)
Proportional hazards (PH) model^1^						
Rectal cancer patients vs comparator	1.21 (1.15–1.28)	3.35 (3.12–3.61)	1.05 (0.96–1.15)	6.43 (5.72–7.22)	3.78 (3.15–4.54)	1.37 (1.24–1.50)
Non-PH model, year of follow-up^2^						
(0–1)	1.81 (1.72–1.91)	5.63 (5.20–6.11)	0.87 (0.80–0.95)	9.51 (8.46–10.70)	6.29 (5.40–7.32)	1.66 (1.51–1.82)
(1–2)	1.14 (1.07–1.21)	3.78 (3.47–4.13)	0.95 (0.87–1.04)	7.02 (6.18–7.97)	3.36 (2.72–4.14)	1.22 (1.10–1.35)
(2–3)	1.10 (1.02–1.18)	3.18 (2.87–3.52)	1.08 (0.98–1.19)	6.03 (5.25–6.93)	3.35 (2.70–4.17)	1.19 (1.05–1.35)
(3–4)	1.06 (0.98–1.14)	2.88 (2.60–3.19)	1.14 (1.03–1.27)	5.36 (4.62–6.22)	3.49 (2.74–4.46)	1.22 (1.07–1.38)
(4–5)	0.97 (0.90–1.05)	2.31 (2.06–2.58)	1.17 (1.04–1.31)	5.30 (4.51–6.22)	2.71 (2.07–3.58)	1.19 (1.04–1.36)
(5–6)	1.00 (0.91–1.09)	2.38 (2.09–2.71)	1.14 (1.01–1.29)	4.86 (4.09–5.78)	2.99 (2.07–4.31)	1.30 (1.11–1.53)
(6–7)	0.95 (0.87–1.05)	2.26 (1.95–2.61)	1.10 (0.96–1.27)	4.65 (3.87–5.60)	2.76 (1.97–3.88)	1.33 (1.01–1.74)
(7–8)	0.96 (0.86–1.07)	2.11 (1.75–2.55)	1.12 (0.95–1.33)	4.82 (3.89–5.98)	2.85 (1.83–4.44)	1.57 (1.14–2.17)
(8–9)	1.00 (0.89–1.13)	2.24 (1.86–2.70)	1.15 (0.95–1.39)	4.59 (3.59–5.86)	2.87 (1.67–4.96)	1.40 (1.13–1.72)
(9–10)	0.95 (0.82–1.10)	1.93 (1.56–2.38)	1.05 (0.86–1.29)	4.61 (3.51–6.06)	2.60 (1.53–4.42)	1.48 (1.19–1.83)
*P*-value for interaction (non-proportional hazards)	< 0.001	< 0.001	< 0.001	< 0.001	< 0.001	< 0.001

^1^The models included adjustment for education level, Charlson Comorbidity Index (CCI), calendar period of diagnosis, age at diagnosis, and sex

^2^The reference level was set to be the comparator group in each follow-up interval

Among the bowel movement–regulating drugs, the rate of constipation medication use was threefold increased (IRR, 3.35; 95% CI, 3.12–3.61) in patients compared with comparators. Similarly, the use of drugs for functional gastrointestinal disorders showed a fourfold increase (IRR, 3.78; 95% CI, 3.15–4.54), and drugs related to diarrhea showed a sixfold increase (IRR, 6.43; 95% CI, 5.72–7.22). Drugs pertaining to acid-related disorders and vitamin or mineral supplements showed a modest increase of 21% (IRR, 1.21; 95% CI, 1.15–1.28) and 37% (IRR, 1.37; 95% CI, 1.24–1.50), respectively. Apart from acid-related disorders, the higher dispensing rate observed in patients remained for the entire follow-up (Table 2).

However, with the exception of constipation and diarrhea, the absolute difference in mean DDDs between patients and comparators was small for several categories and corresponded only to a few DDDs per year (Fig. 3). With respect to temporal trends in dispensing patterns, there was a general tendency toward a higher dispensing rate for acid-related disorder and diabetes drugs in the most recent calendar period (2013–2016) compared to the earliest period (2005–2008) (sTable 2). This trend was further modified among the patients (sTable 2) and interaction analyses (*p*_interaction_ < 0.001) showed a 42% higher dispensing rate in acid-related disorder drugs in the latter period, compared to the early period (IRR, 1.42; 95% CI, 1.27–1.60), and a 22% increased rate of constipation drugs (IRR, 1.22; 95% CI, 1.07–1.40). Drugs used for functional gastrointestinal disorders were dispensed less frequently across the study period among the comparators but were dispensed at a similar rate among patients (*p*_interaction_ < 0.001), while for vitamins and minerals, this pattern was reversed. For drugs used to treat diarrhea, there was no evidence of effect modification of dispensing patterns across calendar time.

**Fig. 4:**
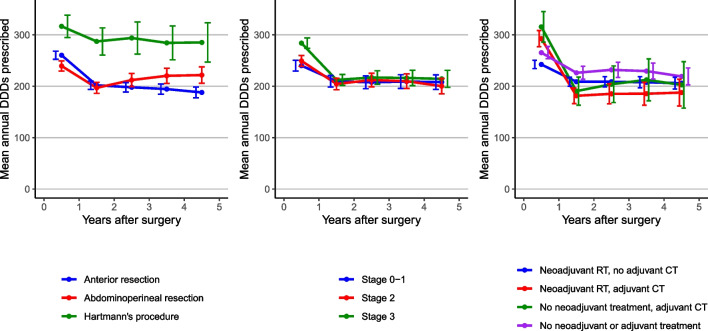
The mean annual defined daily doses (DDDs) of drugs relating to the digestive system (ATC main classification “A”) prescribed to rectal cancer patients diagnosed between 2005 and 2016. Results are presented by surgery type, pathological stage, and oncological treatment group by year of follow-up in relation to the date of curative abdominal surgery. The graphs are not adjusted by covariate factors

The dispensing patterns of digestive drugs by treatment and stage are shown descriptively in Fig. 4 and summarized formally in Table 3.

**Table 3:** Incidence rate ratio (IRR) of defined daily doses of drugs relating to the digestive system estimated from negative binomial regression models. Rectal cancer patients are contrasted by surgical procedure, stage, and oncological treatment

Clinical subgroup	IRR (95% CI), unadjusted	IRR (95% CI), adjusted^1^
Surgical procedure		
Anterior resection	1.00 (reference)	1.00 (reference)
Abdominoperineal resection	1.03 (0.97–1.09)	0.97 (0.91–1.03)
Hartmann’s procedure	1.39 (1.27–1.52)	1.15 (1.05–1.27)
Stage		
0–I	1.00 (reference)	1.00 (reference)
II	1.00 (0.94–1.07)	1.00 (0.93–1.07)
III	1.07 (1.00–1.14)	1.10 (1.03–1.17)
Treatment		
Neoadjuvant RT, no adjuvant CT	1.00 (reference)	1.00 (reference)
Neoadjuvant RT, adjuvant CT	0.97 (0.89–1.05)	1.08 (0.99–1.17)
No neoadjuvant treatment, adjuvant CT	1.05 (0.92–1.20)	1.04 (0.92–1.19)
No treatment	1.09 (1.03–1.16)	0.96 (0.90–1.03)

^1^The adjusted models included education level, Charlson Comorbidity Index (CCI), calendar period of diagnosis, age at diagnosis, and sex

Among the surgical procedures, the rate of prescribed digestive drugs after abdominoperineal resection was similar to that after anterior resection (IRR, 0.97; 95% CI, 0.91–1.03), but treatment with Hartmann’s procedure was associated with a higher dispensing rate of these drugs (IRR, 1.15; 95% CI, 1.05–1.27). However, when omitting DDDs from drugs used in diabetes treatment from the outcome definition, a systematic difference in drug use by operation type was no longer observed (sTable 1, sFig. 1). We observed no systematic difference in drug use pattern when comparing oncological treatment groups in the adjusted models (Table 3).

For stage, there was no significant difference when comparing stage II to stages 0–I (IRR_adjusted_, 1.00; 95% CI, 0.93–1.07), but there was a 10% higher dispensing rate in patients with stage III rectal cancer (IRR, 1.10; 95% CI, 1.03–1.17) compared to the stage 0–I group. The difference was most pronounced in the diagnostic year (Fig. 4).

## Discussion

In this large population-based study investigating drug use among curatively treated rectal cancer patients, we describe how postsurgical morbidity manifests in terms of higher overall drug use in the first year after treatment compared to the general population. We also show excess drug use in the long term (up to 10 years after treatment), primarily of drugs used in regulating digestive symptoms that hint to the potentially prophylactic treatment used in these survivors. Of the results presented by clinical subgroups, as determined by pathological stage, surgical operation types, and oncological treatment, only the advanced stage remained associated with higher digestive drug use after adjustment for diabetic drug use.

Quality of life (QoL) studies after colorectal cancer have previously shown that 30–40% of patients experience impaired QoL 1 year after diagnosis, with reported symptoms including constipation and diarrhea [4, 19]. Long-lasting (up to 10 years) symptoms related to gastrointestinal symptoms after low anterior resection syndrome (LARS) have also been reported consistently in the previous literature [20–22] and are usually the reason behind reports of severe bowel dysfunction, including incontinence, urgency, and frequent bowel movements [23]. The presence of major LARS is thus associated with an inferior quality of life compared with rectal cancer survivors without LARS. Recent studies have shown that LARS may be present in patients treated with anterior resection for rectal cancer even long-term and that the impaired QoL in these patients persists over time [24, 25].

In the present study, drugs for constipation were prescribed at a threefold higher rate to rectal cancer survivors than to the general population, and drugs for diarrhea were prescribed at a sixfold higher rate across the 10-year follow-up period. The fact that excess drug dispensing is driven by drugs associated with the digestive system is, on the one hand, reassuring in the sense that on a population level, symptoms that require pharmacological intervention are limited. On the other hand, we also show that those symptoms that require medical attention and that have previously been reported to be associated with QoL remain for many years. While there was some evidence of effect modification of dispensing rates of drugs that regulate the digestive system across the study period, the temporal trends in patients and comparators were inconsistent across digestive drug subcategories. For example, among patients, higher dispensing rates were observed in the most recent period (2013–2016) for acid-related disorders and constipation whereas vitamins and minerals were dispensed at a lower rate during the same period. Drugs for the treatment of diabetes, diarrhea, and functional gastrointestinal disorders were dispensed at a similar rate between 2005 and 2016.

While patients who had undergone Hartmann’s procedure demonstrated higher prescription rates of digestive system drugs, it should be noted that the increased rates associated with surgical type are likely due to the selection of patients (by age and comorbid conditions at diagnosis) for this treatment [26]. When disregarding drugs associated with diabetes in our data, there was no longer a difference in dispensing rates between treatment groups, which strengthens the suspicion of confounding by indication. The lack of difference in the dispensing of bowel-regulating drugs in patients with a permanent stoma from the start (i.e., after abdominoperineal or Hartman’s resection) and those with anterior resection was somewhat surprising. However, it is likely that the association between bowel dysfunction and quality of life (and thereby excess drug use) is greater in patients with bowel continuity (i.e., after anterior resection). Moreover, following anterior resection, the patients may choose further surgery to receive a permanent stoma if the LARS symptoms are too severe.

In a recent Swedish study including 925 patients with stage I–III colorectal cancer, clinically relevant impairment in global QoL compared to a reference population was reported, with anxiety being most strongly associated with reduced QoL [4]. Another population-based study from Sweden also found that patients diagnosed with colorectal cancer had an increased risk of common mental disorders and related drug use [27]. Our study supports these findings by showing descriptively that the use of drugs for the nervous system in rectal cancer survivors was higher compared to comparators during the initial 2 years after treatment, with the difference gradually disappearing over time. A large proportion of rectal cancer patients have posttreatment symptoms such as fatigue, negative feelings, and psychological hardship following treatment [28, 29]. Drugs for treating fatigue are generally lacking, making drug usage a suboptimal proxy for addressing this potential long-term concern. The observed higher mean DDD of nervous system drug usage during the first couple of years of follow-up might suggest that these symptoms decrease over time. In general, patients worry about relapse during the first years of follow-up, an anxiety that in most cases decreases after the first cancer check-ups at 1 and 3 years after surgery. Further studies on specific psychiatric health aspects in rectal cancer survivors associated with clinical and treatment characteristics are needed to confirm these findings and gain more knowledge.

To the best of our knowledge, this is the first study to perform a comprehensive evaluation of drug use in posttreatment relapse-free rectal cancer patients based on prospectively recorded population-based data while simultaneously controlling for prediagnostic comorbidities. The matched study design and use of health register data have ensured an objective measure of the medical needs among rectal cancer patients treated with surgery. Prescription drug use is an important component of overall healthcare utilization and an indication of patient morbidity and previous reports of sick leave and disability pension [8–10]. However, it can be influenced by factors other than true medical reasons [30]. Sweden has a largely tax-funded healthcare system that ensures that every inhabitant has equal access to healthcare services, including prescribed drugs. For example, in 2022, the annual high cost threshold system in place for medicinal prescriptions was SEK 2350 (approximately 215 USD), after which prescribed drugs are fully subsidized for all citizens [31]. Therefore, the drug prescriptions in Sweden that have been investigated presently tend to reflect the true needs of rectal cancer patients rather than being affected by insurance coverage or other economic reasons [32].

One weakness in our study is that the Prescribed Drug Register only records the dispensed drug and that the amount of the drug that people actually use might differ from the recorded amount, which can lead to misclassification of the outcome. However, this weakness was mitigated by applying a matched cohort design that used individually matched population comparators to provide valid estimates of excess drug use (assuming similar drug-taking behavior among patients and the comparators). Another weakness is that the entire long-term sequelae among rectal cancer survivors might not be fully captured through drug use.

In conclusion, long-term drug use overall in rectal cancer survivors mirrors that of comparators, indicating that these patients are doing quite well in the long run. However, the increased consumption of bowel-regulating drugs in rectal cancer survivors remained up to 10 years after surgery, suggesting persistent bowel dysfunction after rectal cancer treatment.

## Supplementary Information

Below is the link to the electronic supplementary material.

Supplementary file1 (DOCX 14 KB)

Supplementary file2 (DOCX 18 KB)

Supplementary file3 (PDF 11 KB)

## Data Availability

Individual-level data used for the analysis in this study cannot be shared with others in accordance with EU’s GDPR and Swedish research legislation.
