# Supplementary material for: Long-term prescribed drug use in stage I–III rectal cancer patients in Sweden, with a focus on bowel-regulating drugs after surgical and oncological treatment
Source: J Cancer Surviv. 2024 Feb 6;19(4):1244–54. doi: 10.1007/s11764-024-01548-9 (PMC12283852; doi:10.1007/s11764-024-01548-9)
Supplement: Supplementary file 1 — Supplementary file1 (DOCX 14 KB) [file 11764_2024_1548_MOESM1_ESM.docx]

**Supplementary table 1:** Incidence rate ratio (IRR) of Defined Daily Doses (DDDs) of drugs relating to the digestive system estimated from negative binomial regression models. In these analyses drugs used for treatment of diabetes were omitted from the outcome when contrasting clinical subgroups of rectal cancer patients by surgical procedure, stage and oncological treatment.

| **Clinical subgroup** | **IRR (95% CI)**  **Unadjusted** | **IRR (95% CI)**  **Adjusted^1^** |
| --- | --- | --- |
| **Clinical subgroup** | **IRR (95% CI)** | **IRR (95% CI)** |
| **Surgical procedure** |  |  |
| Anterior resection | 1.00 (reference) | 1.00 (reference) |
| Abdominoperineal resection | 0.88 (0.83-0.94) | 0.81 (0.76-0.86) |
| Hartmann´s procedure | 1.29 (1.19-1.41) | 1.03 (0.94-1.13) |
| **Stage** |  |  |
| 0-I | 1.00 (reference) | 1.00 (reference) |
| II | 1.02 (0.95-1.10) | 1.02 (0.95-1.10) |
| III | 1.10 (1.03-1.17) | 1.13 (1.06-1.21) |
| **Treatment** |  |  |
| Neoadjuvant RT, no adjuvant CT | 1.00 (reference) | 1.00 (reference) |
| Neoadjuvant RT, adjuvant CT | 0.97 (0.89-1.05) | 1.09 (1.00-1.18) |
| No Neoadjuvant treatment, adjuvant CT | 0.98 (0.86-1.11) | 0.99 (0.88-1.12) |
| No treatment | 1.10 (1.03-1.17) | 0.97 (0.91-1.03) |

^1^The adjusted models included education level, Charlson Comorbidity Index (CCI), calendar period of diagnosis, age at diagnosis, and sex.
