# Supplementary material for: Long-term prescribed drug use in stage I–III rectal cancer patients in Sweden, with a focus on bowel-regulating drugs after surgical and oncological treatment
Source: J Cancer Surviv. 2024 Feb 6;19(4):1244–54. doi: 10.1007/s11764-024-01548-9 (PMC12283852; doi:10.1007/s11764-024-01548-9)
Supplement: Supplementary file 2 — Supplementary file2 (DOCX 18 KB) [file 11764_2024_1548_MOESM2_ESM.docx]

**Supplementary table 2:** Temporal trends in Incidence rate ratios (IRR) of Defined Daily Doses among rectal cancer patients diagnosed in Sweden between 2005 and 2016 and their matched comparators. A negative binomial regression model was estimated stratified by therapeutic subgroup of digestive drugs and effect modification by case/comparator status was formally tested using likelihood ratio tests.

|  | **Therapeutic subgroup of drugs used for the digestive system** | | | | | | | | | | | |
| --- | --- | --- | --- | --- | --- | --- | --- | --- | --- | --- | --- | --- |
|  | **Acid-related disorders** | | **Constipation** | | **Diabetes** | | **Diarrhea** | | **Functional gastrointestinal disorders** | | **Vitamins and minerals** | |
|  | **IRR (95% CI)** | | **IRR (95% CI)** | | **IRR (95% CI)** | | **IRR (95% CI)** | | **IRR (95% CI)** | | **IRR (95% CI)** | |
| **Main effects model^1,2^** |  |  |  |  |  |  |  |  |  |  |  |  |
| 2005-2008 | 1.0 (ref) |  | 1.0 (ref) |  | 1.0 (ref) |  | 1.0 (ref) |  | 1.0 (ref) |  | 1.0 (ref) |  |
| 2009-2012 | 1.09 (1.05-1.13) |  | 0.94 (0.90-0.99) |  | 1.11 (1.05-1.17) |  | 0.95 (0.89-1.02) |  | 0.88 (0.80-0.97) |  | 0.89 (0.85-0.94) |  |
| 2013-2016 | 1.12 (1.07-1.18) |  | 0.86 (0.82-0.91) |  | 1.12 (1.04-1.20) |  | 1.08 (0.99-1.19) |  | 0.69 (0.61-0.78) |  | 0.92 (0.86-0.98) |  |
| **Interaction model^3^** | **Comparators** | **Cases** | **Comparators** | **Cases** | **Comparators** | **Cases** | **Comparators** | **Cases** | **Comparators** | **Cases** | **Comparators** | **Cases** |
| 2005-2008 | 1.0 (ref) | 1.0 (ref) | 1.0 (ref) | 1.0 (ref) | 1.0 (ref) | 1.0 (ref) | 1.0 (ref) | 1.0 (ref) | 1.0 (ref) | 1.0 (ref) | 1.0 (ref) | 1.0 (ref) |
| 2009-2012 | 1.08 (1.03-1.12) | 1.17 (1.06-1.29) | 0.94 (0.90-0.99) | 0.97 (0.87-1.08) | 1.09 (1.03-1.15) | 1.24 (1.08-1.43) | 0.93 (0.87-1.01) | 1.09 (0.91-1.30) | 0.90 (0.81-0.99) | 0.78 (0.62-1.00) | 0.90 (0.86-0.95) | 0.82 (0.72-0.93) |
| 2013-2016 | 1.07 (1.01-1.13) | 1.42 (1.27-1.60) | 0.80 (0.75-0.85) | 1.22 (1.07-1.40) | 1.13 (1.05-1.22) | 1.05 (0.89-1.24) | 1.04 (0.94-1.15) | 1.35 (1.09-1.68) | 0.64 (0.56-0.73) | 0.99 (0.74-1.31) | 0.95 (0.88-1.01) | 0.76 (0.65-0.88) |
| P-value for interaction | <0.001 | | <0.001 | | =0.0482 | | =0.0617 | | <0.001 | | =0.0251 | |

^1^The models included additional adjustement for education level, Charlson Comorbidity Index (CCI), age at diagnosis, and sex.

^2^A common trend assumed for cases and comparators (i.e. no interaction between calendar time and case/comparator status).

^3^The reference level was parameterized such that the temporal trends are compared with the earliest calendar period for both cases and comparators.
