# Supplementary figures and images for: Long-term prescribed drug use in stage I–III rectal cancer patients in Sweden, with a focus on bowel-regulating drugs after surgical and oncological treatment
Source: J Cancer Surviv. 2024 Feb 6;19(4):1244–54. doi: 10.1007/s11764-024-01548-9 (PMC12283852; doi:10.1007/s11764-024-01548-9)

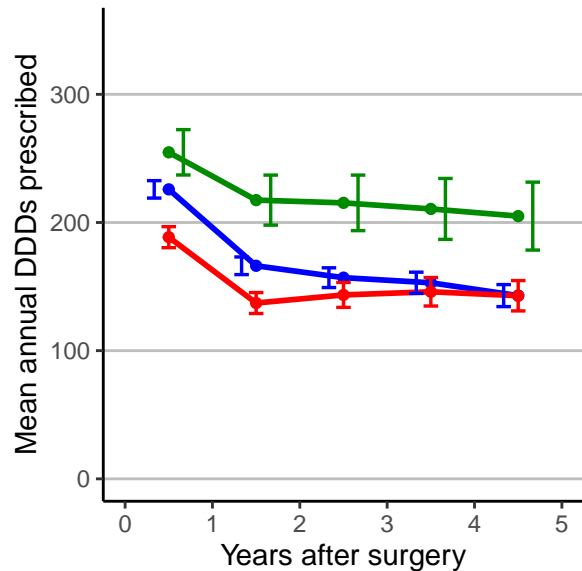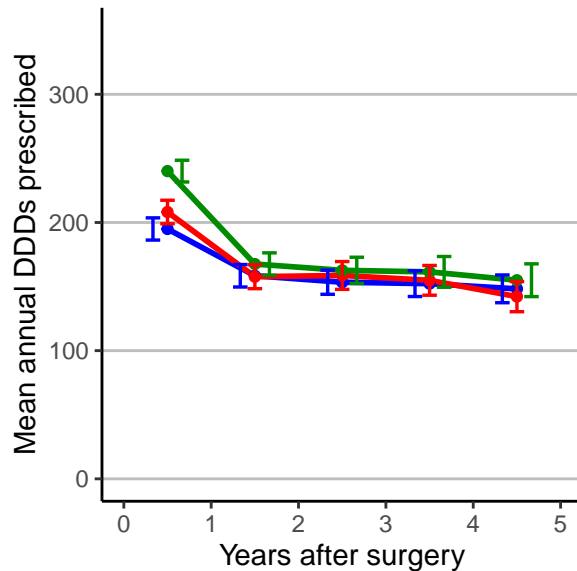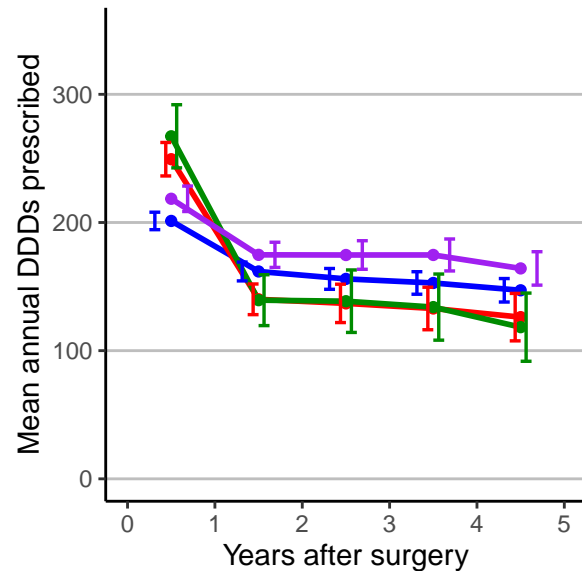

Supplement: Supplementary file 3 — Supplementary file3 (PDF 11 KB) [file 11764_2024_1548_MOESM3_ESM.pdf]
